# Optimized protocols for culturing and sectioning mouse intestinal organoids: enhancing efficiency and structural integrity

**DOI:** 10.1016/j.mex.2026.104016

**Published:** 2026-06-25

**Authors:** Jiawei Li, Rong Jin, Xuanxuan Zhang, Yuecong Chen, Huihan Ai, Xianglin Mei, Ying Sun, Lihua Zheng, Guannan Wang, Yongli Bao, Xiaoli Li

**Affiliations:** a International Joint Research Center of Stem Cell Bank, Ministry of Science and Technology, Northeast Normal University, Changchun, 130117, China; b National Engineering Laboratory for Druggable Gene and Protein Screening, Northeast Normal University, Changchun, 130117, China; c Department of General Surgery, Affiliated Tumor Hospital of Zhengzhou University, Zhengzhou, Henan Province, 450000, China; d Department of Pathology, The Second Hospital of Jilin University, Changchun, 130041, China

**Keywords:** Organoids, Crypt, Embedding and sectioning

## Abstract

Mouse intestinal organoids are essential 3D models that retain host genetic characteristics and complex architecture, making them invaluable for intestinal biology research. However, traditional cultivation and histological processing often suffer from high operational complexity and sample loss during sectioning. This protocol provides an optimized workflow for the establishment of mouse intestinal organoid cultures and their subsequent preparation for immunohistochemistry and fluorescence staining. By refining the handling steps, the method significantly reduces total operational time while enhancing experimental quality. Furthermore, the protocol addresses common challenges in structural integrity, ensuring that the delicate crypt-villus morphology remains intact during the sectioning process. This comprehensive approach offers a robust framework for high-quality histological analysis, supported by a comparative evaluation with traditional methods to guide researchers in different experimental contexts.

• Introduces a direct pellet-OCT embedding strategy that minimizes organoid transfer and sample loss during histological processing.
• Enables high-quality frozen sections compatible with H&E, Alcian Blue, immunohistochemistry, and immunofluorescence staining.
• Provides a practical alternative to conventional agarose-paraffin workflows while preserving organoid morphology and reducing processing complexity.

## Graphical abstract

**Figure:**
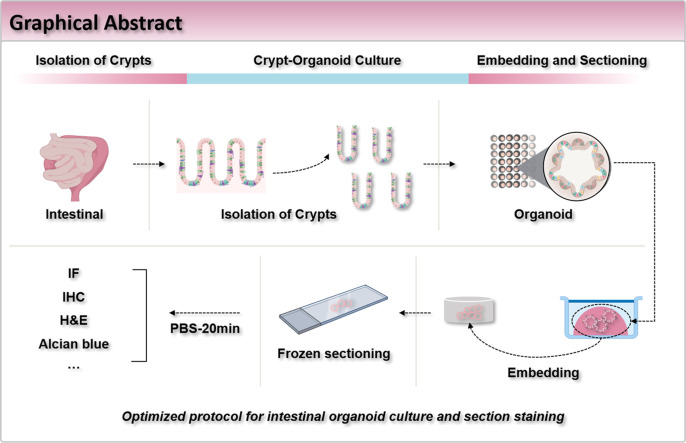

**Specifications table**

**Table:** 

**Subject area**	Biochemistry, Genetics and Molecular Biology
**More specific subject area**	Intestinal organoids;Sectioning and staining
**Name of your method**	Efficient & Structural Intestinal Organoid Culture and sectioning
**Name and reference of original method**	Sato T, Vries RG, Snippert HJ, et al. Single Lgr5 stem cells build crypt-villus structures in vitro without a mesenchymal niche. Nature 2009; 459: 262–265. 2009/03/31. https://www.nature.com/articles/nature07935
**Resource availability**	see Method Details for further information

## Background

Intestinal organoids have revolutionized biomedical research as sophisticated three-dimensional in vitro models that faithfully recapitulate the structural complexity, cellular heterogeneity, and functional properties of native intestinal tissue [[1], [2], [3], [4]]. These self-organizing systems provide unprecedented opportunities to study intestinal development, disease pathogenesis, and drug responses in a physiologically relevant context. Recent advancements in murine intestinal stem cell-derived organoid technology have expanded their utility beyond traditional modeling the crypt-villus differentiation axis to include host-microbe interactions and immune microenvironments through co-culture systems [5,6], offering new perspectives for precision and regenerative medicine [[7], [8], [9]].

Since the pioneering work of Sato et al. in establishing the first intestinal organoid culture system in 2009 [10], the field of intestinal organoid culture has witnessed significant progress, with numerous studies building upon this foundational research [[11], [12], [13]]. Nevertheless, current organoid culture systems suffer from inter-batch variability, limited stability, and reliance on specialized equipment. These limitations often lead to inconsistent results and reduced reproducibility. hindering the widespread application of organoid technology. Furthermore, accurate characterization using immunohistochemistry (IHC) and immunofluorescence (IF) is essential for understanding their biological properties and functions [14,15]. However, the three-dimensional Matrigel embedding of organoids prevents the use of conventional histological techniques. The small size, fragility, and matrix embedding of organoids complicate standard IHC/IF processes, including dehydration, paraffin embedding, and sectioning, thereby increasing the risks of sample loss, structural damage, and procedural complexity.

Building on our previous work [16,17], we developed a streamlined protocol that addresses two major technical bottlenecks in intestinal organoid research: efficient crypt-derived organoid establishment and reliable histological processing of organoids. Unlike conventional workflows that frequently involve agarose embedding, paraffin processing, multiple transfer steps, or technically demanding whole-mount staining procedures, the present protocol combines optimized crypt isolation with a direct pellet-OCT embedding strategy that minimizes sample manipulation and organoid loss.

Importantly, this protocol enables routine preparation of frozen organoid sections that are compatible with H&E, Alcian Blue, immunohistochemistry, and immunofluorescence staining while preserving structural integrity and reducing background interference. By reducing handling complexity and improving sample retention, this workflow provides a practical and reproducible alternative for laboratories performing intestinal organoid-based studies.

## Method details

**Fig. 1:**
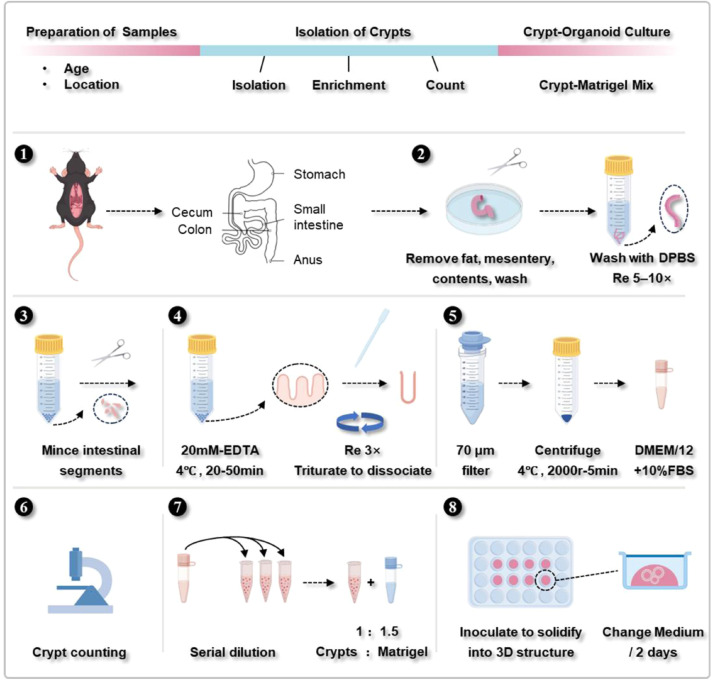
Overview of Crypt-Organoid Optimization Steps. First row: Schematic representation of the three key stages in mouse intestinal organoid generation: (1) intestinal sample preparation, (2) crypt isolation, and (3) organoid culture. Second row (Steps 1–2): Procedure for intestinal sample preparation in organoid culture. Third row (Steps 3–5): Process of isolating intestinal crypts. Fourth row (Steps 6–8): Plating and culture of crypt-Matrigel mixtures.

This protocol is meticulously designed to streamline the process of establishing and culturing mouse intestinal organoids, ensuring optimal outcomes (Fig. 1).

Prior to experimentation, key factors including mouse strain, sex, postnatal age, and intestinal segment (duodenum, jejunum, ileum, colon) must be carefully evaluated, as they directly influence the morphological and functional variability of intestinal organoids. Notably, postnatal age and intestinal origin exert more pronounced effects: organoids derived from young mice exhibit faster growth kinetics and higher differentiation potential, whereas those from older mice display slower growth kinetics, more mature differentiation states, and reduced regenerative capacity [18,19]. Moreover, small intestine and colon organoids inherently differ in structural architecture, culture conditions, functional characteristics, and underlying biological mechanisms [20,21]. Thus, to ensure reliable experimental outcomes, experimental animals, intestinal segments, and culture media formulations must be tailored to specific research objectives.

### Preparation of intestinal samples (Fig. 1, step 1–2)

Example using 8-week-old C57BL/6 male mouse small intestine (duodenum):

After being anesthetized with isoflurane, mice were euthanized by cervical dislocation, submerged in 75% ethanol for surface sterilization, and then transferred to a clean bench for intestinal tissue dissection.

#### Tissue excision

Excise a 10 cm segment of small intestine—starting at the gastric pylorus and including the duodenum—from two 8-week-old male C57BL/6 mice. Extraluminal preparation: Using fine forceps, carefully dissect and remove all connective and adipose tissues to ensure a debris-free culture background. Intraluminal cleaning: Place the tissue on ice in a sterile dish, open the lumen longitudinally with micro-scissors, and flush repeatedly with 10 mL of ice-cold d-PBS (4 °C) using a sterile 10 mL disposable pipette until no fecal debris is visible (Fig. 1, Step 1–2, Tables 1, 2).

**Table 1:** List of reagents for intestinal sample preparation.

Reagents	Cat. no.	Note
Animal	-	Species, age, sex
Isoflurane	RWD -R510–22–10	Anesthetized mice
75% ethanol	-	Sterilization
Ice	-	Protect tissues and cells
DPBS	Stem cell-37,350	Clean & stabilize env

**Table 2:** List of equipment for intestinal sample preparation.

Equipment	Cat. no.	Note
Clean bench	-	-
Beaker	-	+75% ethanol sterilization
Ice - bath dissection board	-	Intestinal Tissue Dissection
Sterile Petri dish	-	Intestinal Tissue Dissection
Sterile surgical instruments (scissors, forceps)	-	For each set/group, avoid cross - use
Sterile 50 mL centrifuge tube	Corning-CLS430829	Wash the intestinal tissue
Sterile disposable plastic pipette	Biosharp	Wash the intestinal tissue

#### Tissue washing

Transfer the cleaned intestinal segments to a 50 mL centrifuge tube. Add 20 mL ice-cold d-PBS, vortex vigorously for 10–15 s, then carefully decant the supernatant to retain tissue. Repeat this wash cycle 5–10 times with fresh 20 mL d-PBS until the effluent is clear and free of particulate matter (Fig. 1, Step 2, Tables 1, 2).

### Isolation of crypts (Fig. 1, step 3–5)

#### Replace the 50 mL centrifuge tube

With forceps, grasp the intestinal segment at the tube mouth and trim it into 1–2 cm pieces, allowing them to drop directly into the fresh tube (Fig. 1, Step 3, Tables 3, 4).

**Table 3:** List of reagents for crypt isolation.

Reagents	Cat. no.	Note
DPBS	Stem cell-37,350	Clean & stabilize env
20 mM EDTA (DPBS)	Sigma-03,690	Dissociation of crypts
DMEM/F12+10%FBS	Stem cell-36,254 DMED/F12	Terminate the action of EDTA

**Table 4:** List of equipment for crypt isolation.

Equipment	Cat. no.	Note
Sterile surgical instruments	-	Scissors, Forceps (set/group)
Sterile 50 mL centrifuge tube	Corning-CLS430829	Clean & stabilize env
Sterile disposable plastic pipette	Biosharp	Triturate- dissociate crypts
Sterile 1.5 mL centrifuge tube	-	Enrich crypts
70 μm filter mesh	Corning-352,350	Filter crypts
4 °C centrifuge	-	-
4 °C shaker	-	-

#### Incubate with EDTA-DPBS

Let the segments settle by gravity for 30 s, then carefully decant the supernatant without losing tissue. Add 15 mL of cold 20 mM EDTA-DPBS and incubate at 4 °C with gentle agitation for 40 min (20 min for small intestine; 40 min for colon). Using a sterile disposable pipette, vigorously pipette the suspension up and down 5 times to dissociate crypts. Transfer the supernatant to a new 50 mL centrifuge tube designated ①. Add 10 mL of ice-cold DPBS to the original tube containing residual tissue. Repeat trituration 5 times, allow tissue fragments to settle by gravity for 30 s, and transfer the supernatant to tube ①. Repeat the wash cycle once with fresh 10 mL DPBS, combining all supernatants in tube ① (total volume: ∼30 mL). Note: Retain the original tube with residual segments at 4 °C as a backup for additional crypt enrichment if yield is insufficient (Fig. 1, Step 4, Tables 3, 4).

**Fig. 2:**
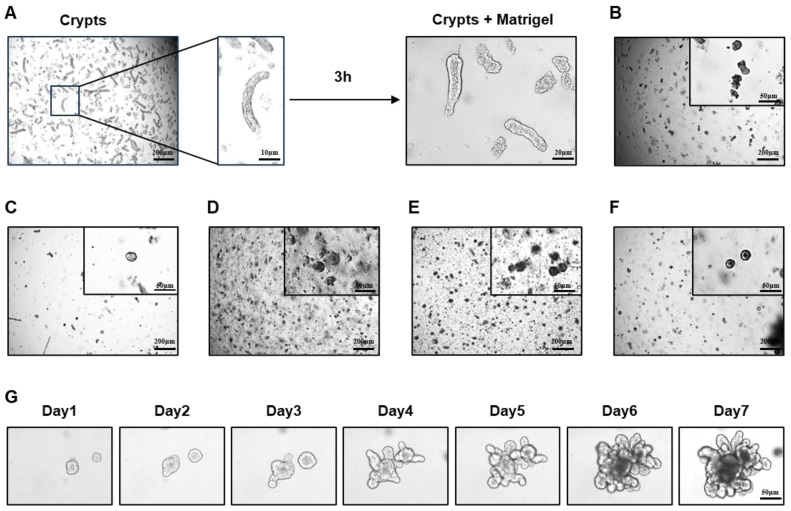
Isolated Crypt and Organoid Culture. A: Left panel: Isolated single small intestinal crypts; Right panel: Organoids formed 3 h after plating crypt-Matrigel mixtures. B: Disintegrated crypt structures due to excessively long EDTA treatment. C: Insufficient crypt seeding density. D: Excessive crypt seeding density. E: High background debris. F: Organoids suitable for continued culture. G: Representative images depicting the morphological evolution of organoids de-rived from 6-week-old mouse small intestine (duodenum) organoids after 7 days of culture.

Critical note: The optimal EDTA incubation time depends on intestinal region, mouse age, and tissue condition. For example, treatment of duodenal tissues from 6-week-old mice with 20 mM EDTA at 4 °C for 20 min typically yields intact crypt structures suitable for organoid culture (Fig. 2A). Excessive EDTA exposure may lead to crypt disintegration and reduced organoid-forming efficiency (Fig. 2B). The optimal duration of EDTA treatment varies according to mouse age, intestinal region (small intestine versus colon), and tissue condition (e.g., inflammation or tumor-associated changes). Therefore, crypt morphology should be periodically assessed during the isolation procedure to avoid over-digestion and crypt fragmentation. Excessive EDTA exposure may compromise organoid-forming efficiency by disrupting crypt integrity (Fig. 2B).

#### Filtration and crypt enrichment

Position a 70 μm filter mesh over a clean 50 mL centrifuge tube with forceps, then pour the pooled supernatant from tube ① through the filter. Centrifuge the filtrate at 4 °C, 2000 rpm for 5 min. Carefully decant the supernatant without disturbing the pellet. Add 1 mL of cold DMEM/F12 supplemented with 10% FBS to neutralize residual EDTA. Resuspend the pellet by gentle pipetting and transfer it to a 1.5 mL microcentrifuge tube (Fig. 1, Step 5, Tables 3, 4).

### Crypt culture – organoids (Fig. 1, step 6–8)

#### Crypt resuspension and counting

Centrifuge at 4 °C, 2000 rpm for 5 min. Thaw Matrigel on ice and prepare organoid culture medium. Discard the supernatant (remove completely using a pipette). Add 500 μL of organoid culture medium, resuspend the pellet by pipetting, then transfer 10 μL to a cell counting chamber to assess crypt isolation efficiency and quantify crypt density. Dilute the suspension to achieve a target density of 20–100 crypts per well in a 24-well plate (∼4–30 crypts per 10 μL) (Fig. 1, Step 6, Table 5–6).

**Table 5:** List of reagents for Crypt culture.

Reagents	Cat. no.	Note
Organoid Growth Medium	Stem Cell 06,005	EGF, Noggin, R-spondin
Matrigel	Corning-356,231	Medium for 3D

**Table 6:** List of reagents for Crypt culture.

Equipment	Cat. no.	Note
37 °C CO₂ incubator	-	Provide a culture env
Pre - cooled pipette tips	-	Aspirate Matrigel (−20 °C)
Pre - warmed 24 - well plate	Corning-3526	Promote Matrigel solidification (37 °C)
Cell counting chamber	-	Count the number of crypts
Microscope	-	Count the number of crypts
Sterile 1.5 mL centrifuge tube	-	Dilute the number of crypts
Pipette	-	Mix

#### Crypt-matrigel mixture preparation

For gradient-density crypt suspensions, combine 20 μL of crypt suspension with 30 μL of Matrigel per well. Use a prechilled pipette tip to aspirate Matrigel, allowing a slight over-volume (≈10%) to compensate for tip adhesion. Transfer gradient-density crypt suspension to a 1.5 mL tube first, then use a fresh prechilled tip to aspirate Matrigel and mix thoroughly (Fig. 1, Step 7, Tables 5, 6).

Note: Pipette the crypt-Matrigel mixture gently and slowly to avoid air bubbles, as rapid pipetting can generate friction with viscous Matrigel. Leaving a small residual volume in the tip to minimize bubble formation.

#### Plating and culture initiation

After mixing, pipette 50–60 μL of the mixture onto the center of a prewarmed (37 °C) 24-well plate to form a droplet. Incubate at 37 °C for 10 min to allow Matrigel solidification. Select wells with clear background and stable organoid structures. Gently add 300 μL of culture medium along the well wall, avoiding disruption of Matrigel droplets (Fig. 1, Step 8, Tables 5, 6).

Critical considerations for organoid culture: Both insufficient and excessive crypt seeding densities negatively affect organoid formation efficiency (Fig. 2C–D). For a 24-well plate, seeding 20–100 crypts per well is recommended. In addition, contaminating debris originating from luminal contents, adipose tissue, or connective tissue may impair organoid growth and increase background interference (Fig. 2E). Therefore, careful tissue cleaning and sufficient washing during crypt isolation are essential. Approximately 3 h after plating, spherical organoids with clear backgrounds and moderate density should be visible (Fig. 2F). These organoids can then be maintained for 5–7 days with medium replacement every 2 days, resulting in mature organoid structures (Fig. 2G). Gently add 300 μL of culture medium along the well wall, avoiding disruption of Matrigel droplets.

### Optimized protocol for organoid embedding, sectioning, and staining (Fig. 3, step 1–4)

The minute dimensions (≈50–300 μm), delicate architecture, and Matrigel-embedded growth of organoids pose significant challenges to traditional agarose-paraffin embedding methods. This conventional approach is plagued by intricate procedures that frequently result in tissue damage or loss. Specifically, dehydration-induced shrinkage of agarose distorts organoid morphology and exacerbates sectioning difficulties, while high-temperature wax infiltration compromises protein conformation. During antigen retrieval for IHC, paraffin-agarose-embedded organoids often detach from slides, undermining assay reliability.

**Fig. 3:**
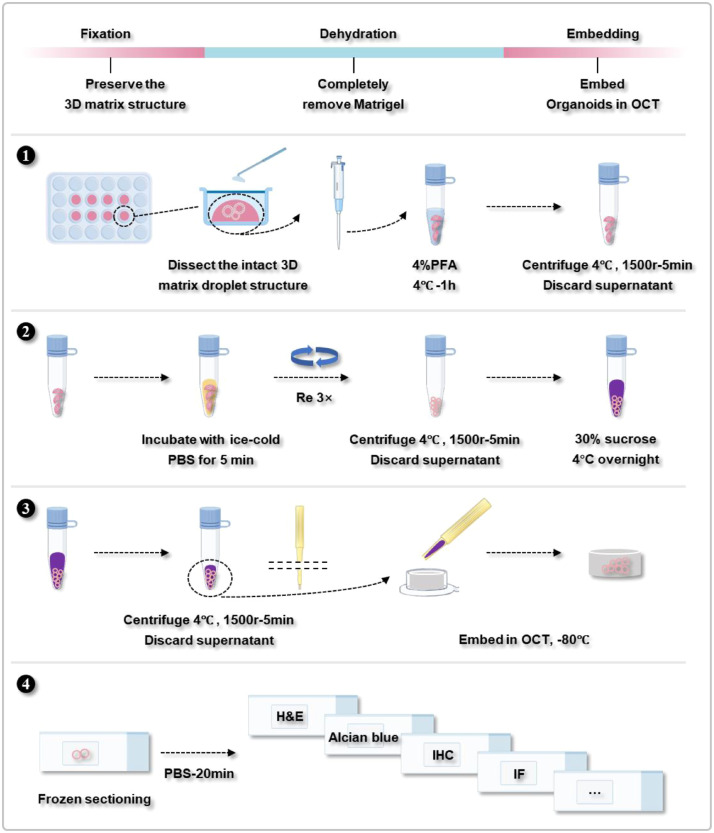
Overview of Optimization Steps for Organoid Embedding, Sectioning, and Staining. First row: Three components of organoid section staining: fixation, dehydration, and embedding. Second row (Steps 1): Organoid fixation process. Third row (Steps 2): Organoid dehydration process. Fourth row (Steps 3): Organoid embedding process. Fifth row (Steps 4): Frozen Sections and Compatible Staining Protocols.

To overcome these limitations, a streamlined embedding protocol is presented here that minimizes organoid manipulation, preserves morphological fidelity, and simplifies workflow. Experimental validation confirms that sections prepared by this method are compatible with a range of staining techniques, including high-temperature antigen retrieval-based IHC, IF, and classic staining assays (e.g., H&E, Alcian blue). These sections exhibit distinct staining patterns with minimal background interference, eliminating the need for gradient ethanol dewaxing—simply soaking in PBS prior to staining suffices (Fig. 3).

#### Exposure and collection of organoids

Discard the culture medium to expose the 3D "water droplet" structure. Using a cell scraper, carefully detach the Matrigel droplet from the culture plate by running the scraper along the bottom edge of the droplet. Transfer the collected mixture (gel-like clumps) to a 1.5 mL microcentrifuge tube using a trimmed pipette tip (cut end ≥2 mm wide). Add 1 mL of pre-chilled 4% paraformaldehyde, then incubate on ice for 1–2 h (recommend pooling organoids from 2 to 3 wells for sufficient yield) (Fig. 3, Step 1, Table 7–8).

**Table 7:** List of reagents for organoid embedding and sectioning.

Reagents	Cat. no.	Note
Pre-cooled 4% paraformaldehyde	Solarbio-P110	Fix the organoids
Pre - cooled PBS	**-**	Wash the Matrigel
Pre - cooled 30% sucrose solution	**-**	Dehydrate the organoids
OCT compound	SAKURA 4583	Embed the organoids

**Table 8:** List of equipment for organoid embedding and sectioning.

Equipment	Cat. no.	Note
Cell scraper	Corning-3010	Collect the organoids
4 °C centrifuge	**-**	Enrich organoids
Pipette	**-**	Transfer
Sterile 1.5 mL centrifuge tube	**-**	Enrich organoids
−80 °C refrigerator	**-**	Store OCT - organoids
Frozen microtome	**-**	Prepare frozen sections
Disposable blade R35	8093,081	Prepare frozen sections
Ion adsorption slide	CITOTEST-188,105	Prepare frozen sections

#### Centrifugation and washing

Centrifuge at 1500 rpm for 5 min at 4 °C. Carefully aspirate the paraformaldehyde, leaving ∼50 μL to avoid disturbing the pellet at the tube bottom. Wash with ice-cold PBS: Add 1 mL PBS, incubate 5 min on ice, centrifuge at 1500 rpm for 5 min, then aspirate supernatant (leave ∼50 μL). Repeat twice more (total 3 washes). Dehydrate with sucrose: Add 1 mL of 30% sucrose solution, incubate overnight at 4 °C (ensure complete penetration) (Fig. 3, Step 2, Table 7–8).

#### Centrifugation and embedding

Centrifuge the suspension at 1500 rpm for 5 min at 4 °C. The sucrose supernatant is partially removed using a pipette, leaving a minimal volume (≈50–100 μL) to cover the organoid pellet. Trim a 200 μL pipette tip at approximately one-third of its length to create an opening diameter of >3 mm. This modification prevents mechanical damage to organoids during aspiration. With the modified tip, gently aspirate the organoid pellet from the tube base. Insert the tip into a 1.5 mL tube cap prefilled with OCT. Slowly dispense the organoid–sucrose suspension into the center of the OCT, avoiding complete tip evacuation and bubble introduction. Freeze immediately: Label the tube, then transfer to –80 °C for ≥1 hour until OCT solidifies (Fig. 3, Step 3, Tables 7, 8).

#### Sectioning and mounting

Prepare frozen sections: After OCT solidification (1–3 h), section at 5–10 μm thickness using a cryostat. Mount on slides: Collect sections on ion-adsorption slides, then store at –20 °C in the dark until staining (Fig. 3, Step 4, Table 7, 8).

#### Organoid section immunofluorescence staining

**Fig. 4:**
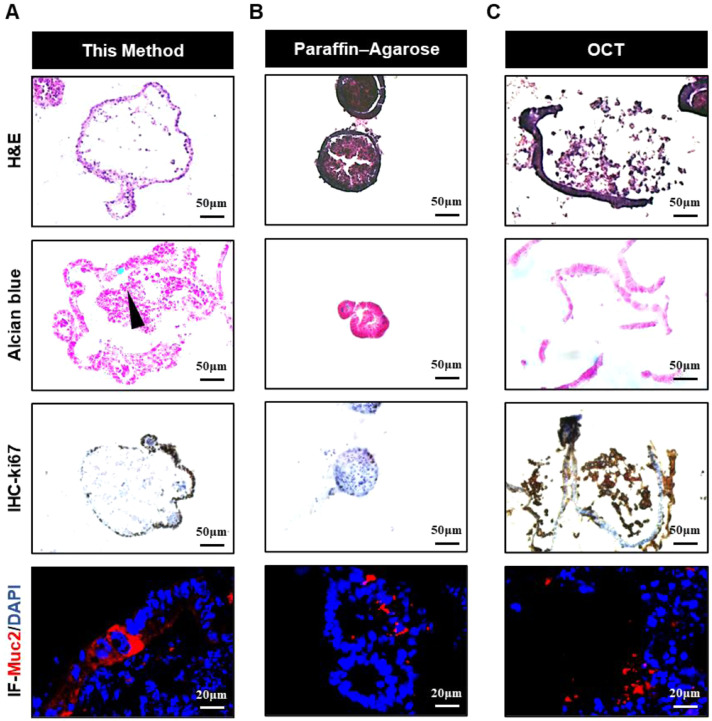
Comparison of Staining Efficacy among Paraffin-Agarose, OCT, and the This Method for HE, Alcian Blue, IHC, and IF Staining. Representative images of HE, Alcian Blue, IHC, and IF staining were obtained using the same batch of cultured mouse intestinal organoids, with all experimental conditions kept identical except for embedding methods. A. This method: Exceptional preservation of organoid tissue architecture was demonstrated, with intact morphological structures maintained in H&E and Alcian Blue staining. Specific antibody binding with clear background was achieved in IHC/IF staining. B. Paraffin-agarose embedding: This approach preserved overall organoid architecture; however, alcohol dehydration and high‑temperature wax infiltration induced agarose shrinkage. Organic solvent treatment masked antigen epitopes, thereby reducing IHC and IF specificity. C. Traditional OCT embedding: Detachment and loss of morphological structures in organoid sections were frequently observed, accompanied by Matrigel residue. Non-specific background staining was elicited during staining procedures due to these factors.

1. Section Rehydration: Allow frozen sections to equilibrate at room temperature (RT, 22–25 °C) for 30 min. Then immerse in PBS for 30 min, replacing the buffer every 15 min.
2. Permeabilization (Optional): The tissue perimeter is circled with a Pap Pen. Permeabilization is performed depending on antigen localization (extracellular proteins typically do not require this step). Sections are incubated in 0.1% Triton X-100 in PBS for 45 min, followed by three washes in PBS (3 min each).
3. Blocking: Block sections in 2% BSA in PBS within a humidified chamber at room temperature for 1 h. Remove excess BSA by gentle aspiration (no wash).
4. Apply primary antibody diluted in PBS at the recommended concentration to the sections. Incubate sections overnight at 4 °C in a humidified chamber.
5. Post-Incubation Washing: Allow sections to equilibrate at room temperature for 1 h. Wash three times in PBS (3 min each) to remove unbound primary antibody.
6. In the dark, apply fluorescent secondary antibody diluted in PBS to the sections. Incubate for 1 h at room temperature, then wash three times in PBS (3 min each).
7. Nuclei Counterstaining: Counterstain nuclei with DAPI in the dark at room temperature for 10 min. Wash twice in PBS (5 min each) to minimize background.
8. Mounting and Storage: Sections were mounted under coverslips with antifade mounting medium to preserve fluorescence. Slides were stored at 4 °C in the dark and imaged within 24 h to prevent photobleaching (Fig. 4A-IF, Table 9).

**Table 9:** List of reagents for organoid Section IF staining.

Reagents	Cat. no.	Note
PBS	-	Clean & stabilize env
Pap Pen	G6100	Liquid Blocker
0.1%Triton X-100	Sigma–9036–19–5	Increase cell memb. perm.
2%BSA	Sigma-10,711,454,001	Block non-specific sites
Primary antibody	-	-
Fluorescent secondary antibody	-	-
Coverslip	CITOTEST-10212424C	-
DAPI	Sigma-D9542	Stain the cell nucleus
AntiFade Mounting Medium	MCE-HY-K1042	-

#### Organoid section IHC staining

1. Section Rehydration: Frozen sections are equilibrated at room temperature for 30 min, then immersed in PBS for 30 min with two changes (every 15 min).
2. Antigen Retrieval (High-Temperature Method): Slide racks are completely submerged in sodium citrate retrieval buffer (pH 6.0). In a pressure cooker, timing starts for 3 min after the air valve emits steady steam. Sections are allowed to cool naturally to RT, followed by three rinses with PBS (5 min each).
3. Peroxidase Blocking: Peroxidase blocker is applied to sections, which are incubated at RT for 10 min. Sections are rinsed twice with PBS (5 min each).
4. Primary Antibody Incubation: Primary antibody, diluted in PBS at the concentration recommended by the manufacturer, is applied directly to the tissue sections. A hydrophobic barrier is created around the tissue using a Pap Pen to prevent solution overflow. Sections are incubated overnight at 4 °C in a humidified chamber. Subsequently, sections are rinsed twice with PBS (5 min each).
5. Stringent Washing: Sections are rinsed three times with PBS (5 min each).
6. Secondary Antibody Conjugate Incubation: Enzyme-labeled goat anti-rabbit/mouse IgG polymer is applied to sections, which are incubated at RT for 20 min. Sections are rinsed three times with PBS (3 min each).
7. DAB Chromogenic Development: Freshly prepared DAB chromogenic solution is applied, and sections are incubated in the dark for 3–5 min. Color development is monitored under a microscope to prevent nonspecific staining, followed by termination with pure water and three PBS rinses (3 min each).
8. Hematoxylin Counterstaining: Sections are counterstained with hematoxylin for 2 min, then rinsed with running water.
9. Dehydration, Clearing, and Mounting: Sections are dehydrated through a gradient of ethanol (70% → 80% → 95% → 100%), cleared with xylene, and mounted with neutral balsam. Slides are imaged under a microscope (Fig. 4A-IHC, Table 10).

**Table 10:** List of Reagents for Organoid Section IHC Staining.

Reagents	Cat. no.	Note
Antigen retrieval solution	-	Select as required
PBS	-	-
Immunohistochemistry Kit	Zsbio	Select as required
DAB Chromogenic Kit	Zsbio	Locate the chromogenic antibody
Hematoxylin	Solarbio-G1080	Nuclear staining
Ethanol	-	Dehydration
Xylene	-	Clearing
Neutral	Biosharp-BL704A	Mounting

#### Organoid section H&E staining

1. Frozen sections are equilibrated at RT for 30 min, then immersed in PBS for 30 min with two changes (every 15 min).
2. Eosin Staining: Sections are stained with eosin for 2 min, then rinsed with running water.
3. Hematoxylin Counterstaining: Sections are counterstained with hematoxylin for 2 min, followed by rinsing with running water.
4. Dehydration, Clearing, and Mounting: Sections are dehydrated through a gradient of ethanol (70% → 80% → 95% → 100%), cleared with xylene, and mounted with neutral balsam. Sections are then imaged under a microscope (Fig. 4A-H&E, Table 11).

**Table 11:** List of reagents for organoid Section H&E staining.

Reagents	Cat. no.	Note
H&E Staining Kit	Solarbio-G1080	-

#### Organoid section alcian blue staining

1. Frozen sections are equilibrated at RT for 30 min, then immersed in PBS for 30 min with two changes (every 15 min).
2. Alcian Blue Staining: Alcian Blue 8GX staining solution is prepared, and sections are incubated in the solution for 30 min. Drops of the stain are added every 10 min to prevent evaporation. Sections are rinsed with running water for 2 min.
3. Nuclear Fast Red Counterstaining: Sections are counterstained with 0.2% nuclear fast red for 15 min, followed by rinsing with running water for 3 min.
4. Dehydration, Clearing, and Mounting: Sections are dehydrated through a gradient of ethanol (70% → 80% → 95% → 100%), cleared with xylene, and mounted with neutral balsam. Sections are then imaged under a microscope (Fig. 4A-Alcian Blue, Table 12).

**Table 12:** List of Reagents for Organoid Section Alcian Blue Staining.

Reagents	Cat. no.	Note
Alcian Blue 8GX	Solarbio–33,864-99–2	
Nuclear Fast Red solution, 0.2%	Solarbio-G1321

Critical parameters throughout the protocol and common troubleshooting strategies are summarized in Table 13. These recommendations are based on repeated optimization experiments and are intended to facilitate successful crypt isolation, organoid culture, embedding, sectioning, and downstream staining procedures.

**Table 13:** Critical parameters, common pitfalls, and troubleshooting strategies for intestinal organoid culture and histological processing.

Step	Problem	Possible Cause	Solution
Tissue washing	High debris background	Incomplete removal of luminal contents, adipose tissue, or connective tissue	Increase washing cycles and carefully remove residual tissue contaminants
EDTA incubation	Low crypt yield	Insufficient EDTA exposure	Extend incubation time according to tissue type and age
EDTA incubation	Crypt fragmentation	Excessive EDTA exposure	Reduce incubation time and monitor crypt morphology during isolation
Crypt seeding	Low organoid formation efficiency	Insufficient crypt density	Increase seeding density to 20–100 crypts per well
Crypt seeding	Organoid fusion or overcrowding	Excessive crypt density	Reduce seeding density
Matrigel plating	Air bubbles in Matrigel	Rapid pipetting	Mix gently using pre-cooled pipette tips
Organoid collection	Sample loss	Excessive transfer steps or narrow pipette tips	Use trimmed wide-bore pipette tips and minimize handling
Embedding	Organoid displacement in OCT	Incomplete pellet enrichment	Carefully concentrate organoids before embedding
IF/IHC staining	High background staining	Residual Matrigel or inadequate washing	Increase PBS washing steps before staining
IHC staining	Weak signal intensity	Antigen damage or insufficient retrieval	Optimize antigen retrieval conditions and antibody dilution

## Method validation

Crypt-villus units in the small intestine and colonic crypts represent key functional units of adult stem cell biology, playing pivotal roles in tissue homeostasis and regeneration [22,23]. Organoid culture systems have emerged as powerful tools to maintain the long-term regenerative potential of stem cells outside their native niche, representing a significant breakthrough for clinical applications [24,25].Since Sato et al. first established intestinal organoid cultures in 2009 [10], these methodologies have been continuously refined. The most robust method remains the Matrigel-based three-dimensional system, in which intact intestinal crypts or purified Lgr5⁺ crypt base columnar (CBC) stem cells are embedded in laminin-rich Matrigel. The matrix is supplemented with a defined cocktail of growth factors essential for stem cell maintenance and proliferation, typically including the BMP inhibitor Noggin, the WNT agonist R-spondin1, epidermal growth factor (EGF), and a Notch ligand [26].

The principal innovation of the present protocol lies not in the organoid culture system itself, which is based on established crypt-derived organoid methodologies, but in the integration of optimized crypt handling procedures with a direct pellet-OCT embedding workflow. This approach reduces sample transfer steps, minimizes organoid loss during processing, and facilitates downstream histological analyses using a single standardized workflow. Our preliminary comparisons showed that preparing customized growth factor cocktails and using commercial pre-optimized mouse/human small intestine and colon organoid media yield comparable experimental outcomes and similar overall costs. Consequently, culture media component sources were not strictly standardized across experiments. Furthermore, rapid advances and wider accessibility of organoid technology in recent years have significantly reduced associated culture costs [27,28]. Considering these factors and the equivalence of culture media, this study prioritized optimizing operational procedures to reduce processing time, lower technical complexity, and improve experimental efficiency.

Organoid IHC and H&E staining conventionally use paraffin–agarose embedding. This conventional approach has several drawbacks. Dehydration can lead to agarose shrinkage and hardening, which in turn distorts the morphology of organoids and complicates the sectioning process. Additionally, the combination of high-temperature wax infiltration and ethanol dehydration often results in organoid loss or structural damage. Here, we present an optimized embedding workflow (Fig. 3). This workflow simplifies the experimental process by reducing steps and minimizing organoid transfer, thereby improving experimental efficiency and effectively eliminating the impact of Matrigel on subsequent section staining. Furthermore, prior to staining frozen sections, a simple PBS incubation removes the OCT, thereby eliminating the need for dewaxing step. Notably, during high-temperature antigen retrieval, organoid sections prepared by this protocol maintain adhesion. The workflow is also compatible with immunofluorescence (IF) and Alcian Blue staining (Fig. 4A).

## Advantages of the present protocol

Compared with conventional intestinal organoid processing workflows, the present protocol offers several practical advantages:

1. Reduced sample manipulation through direct pellet-OCT embedding, thereby minimizing organoid loss.
2. Elimination of agarose embedding and paraffin processing steps, reducing overall processing complexity.
3. Compatibility with multiple downstream applications, including H&E, Alcian Blue, immunohistochemistry, and immunofluorescence staining.
4. Improved preservation of organoid morphology and structural integrity during sectioning.
5. Reduced background interference caused by residual Matrigel and paraffin-processing artifacts.

These advantages make the protocol particularly suitable for laboratories seeking a simple and reproducible workflow for organoid histology.

A summary comparison between the present workflow and commonly used organoid processing approaches is provided in Fig. 4 and Table 14.

**Table 14:** Quantitative comparison of different organoid processing workflows.

Evaluation Parameter	Present Method	Agarose–Paraffin Embedding	Traditional OCT Embedding
Section integrity rate (%)	92	75	83
Tissue detachment rate (%)	5 ± 1	40 ± 8	30 ± 5
Cellular shrinkage rate (%)	< 5	40 ± 6	15 ± 3
Fluorescence signal intensity (AU)	250 ± 20	80 ± 10	120 ± 15

Table 14. Quantitative comparison of histological performance among the present workflow, agarose–paraffin embedding methods, and traditional OCT embedding. Section integrity rate was calculated as the percentage of intact organoid sections retained after sectioning and staining. Tissue detachment rate represents the percentage of sections exhibiting partial or complete tissue loss during staining procedures. Cellular shrinkage rate was determined by comparing organoid dimensions before and after processing. Fluorescence signal intensity was quantified from representative immunofluorescence images under identical imaging conditions and is expressed as arbitrary units (AU).

To quantitatively evaluate the performance of different organoid processing workflows, section integrity, tissue detachment, cellular shrinkage, and fluorescence signal preservation were compared among the present method, agarose–paraffin embedding approaches, and traditional OCT embedding (Table 14). The present workflow exhibited the highest section integrity rate (92%), the lowest tissue detachment rate (5 ± 1%), and minimal cellular shrinkage (<5%). In addition, immunofluorescence signal intensity was substantially higher than that observed with conventional OCT and agarose–paraffin methods, indicating superior preservation of tissue morphology and antigenicity.

## Limitations

Compared with paraffin–agarose embedding, this method is unsuitable for long-term tissue storage. Although non-antigen–dependent stains (e.g., H&E and Alcian blue) remain stable, antigenic epitopes may become inaccessible after storage at –80 °C for over one year.

Time variability: Isolation and culture steps depend on tissue quality and operator skill.

Storage consideration: Frozen sections stored at –80 °C are optimal for staining within 6 months for IHC/IF applications.

## Ethics statements

### Animals and sample collection

Male C57BL/6 mice (8 weeks old) were handled under approval from Northeast Normal University IACUC (NENU-IACUC-2025–011-M). Mice were euthanized by isoflurane overdose and cervical dislocation. A 10 cm segment of small intestine (duodenum) was removed, opened, and rinsed in ice‑cold PBS. Preparation of Intestinal Samples, Protocols for Crypt Isolation, Organoid Culture, Embedding and Sectioning, and Staining Procedures (IF, IHC, H&E, Alcian Blue) are described in full detail in the Results section.

### Institutional review board statement

The study was conducted in accordance with the Declaration of Helsinki. All animal studies were conducted with approval from the Animal Research Ethics Committee of Northeast Normal University (NENU-IACUC-2025–011-M) of China and performed in accordance with established guidelines.

## Declaration of competing interest

The authors declare that they have no known competing financial interests or personal relationships that could have appeared to influence the work reported in this paper.

## Data Availability

Data will be made available on request.
